# Epigenome editing of human hematopoietic stem cells enables sustained and reversible thrombosis prevention

**DOI:** 10.64898/2026.03.27.714536

**Published:** 2026-03-29

**Authors:** Tianyi Ye, Wanying Xu, Maria N. Barrachina, Peng Lyu, Mateusz Antoszewski, Lucrezia della Volpe, Chun-jie Guo, Andrew J. Lee, Madelaine S. Theardy, Spencer D. Shelton, Lara Wahlster, Alexis Caulier, Luana Messa, Michael Poeschla, Gaurav Agarwal, Ronodeep Mitra, Alec A. Schmaier, Jonathan S. Weissman, Kellie R. Machlus, Vijay G. Sankaran

**Affiliations:** 1 Division of Hematology/Oncology, Boston Children’s Hospital, Harvard Medical School, Boston, MA 02115, USA; 2 Department of Pediatric Oncology, Dana-Farber Cancer Institute, Harvard Medical School, Boston, MA 02115, USA; 3 Howard Hughes Medical Institute, Boston, MA 02115, USA; 4 Broad Institute of MIT and Harvard, Cambridge, MA 02142, USA; 5 Harvard Stem Cell Institute, Cambridge, MA 02138, USA; 6 Vascular Biology Program, Boston Children’s Hospital, Boston, MA 02115, USA; 7 Department of Surgery, Boston Children’s Hospital, Harvard Medical School, Boston, MA 02115, USA; 8 Cardiovascular Research Center, Beth Israel Deaconess Medical Center, Boston, MA 02115, USA; 9 Division of Cardiovascular Medicine, Beth Israel Deaconess Medical Center, Boston, MA 02115, USA; 10 Dvision of Hemostasis and Thrombosis, Beth Israel Deaconess Medical Center, Boston, MA 02115, USA; 11 Whitehead Institute for Biomedical Research, Cambridge, MA 02142, USA; 12 Department of Biology and Howard Hughes Medical Institute, Massachusetts Institute of Technology, Cambridge, MA, 02142, USA; 13 David H. Koch Institute for Integrative Cancer Research and Massachusetts Institute of Technology, Cambridge, MA 02142, USA

## Abstract

Thrombosis remains a major cause of cardiovascular and cerebrovascular diseases, driven in large part by platelet activation and aggregation. Because platelets are continuously produced from hematopoietic stem cells (HSCs), durable reprogramming of HSC output offers a unique opportunity for a one-time antithrombotic intervention. Here, we show that DNA methylation-based epigenome editors delivered transiently as RNA result in stable, heritable gene silencing in primary human HSCs that persists through long-term self-renewal and megakaryocytic differentiation, while remaining reversible through targeted demethylation. Targeting the platelet integrin β3 (*ITGB3*), this approach achieves robust, sustained repression and yields platelets with impaired aggregation. Extending this framework to additional genetically-nominated platelet targets establishes HSC epigenome editing as a durable and reversible strategy to modulate thrombotic risk and highlights broader opportunities to engineer hematopoiesis.

## Introduction

Thrombosis, the formation of a clot within blood vessels, is a major cause of life-threatening conditions such as ischemic heart disease and strokes that are estimated to impact over 40 million people around the world each year.^1,2^ A mainstay to prevent and treat these clots involves blockade of platelet activation through the use of antiplatelet therapies, such as aspirin and clopidogrel.^3–6^ Unfortunately, these therapies require lifelong daily administration, which is often complicated by poor compliance and some patients can develop resistance to these therapies, limiting the effectiveness of clot prevention.^7,8^ Moreover, platelet function studies have shown that even among patients receiving standard antiplatelet therapy, a subset have persistently high on-treatment platelet reactivity, which is associated with an increased risk of recurrent thrombotic events.^9,10^ Therefore, there is a need to develop improved antiplatelet therapies that are durable and broadly effective.

Platelets, as with all other blood cells in the circulation, originate from rare hematopoietic stem cells (HSCs) that reside within the bone marrow and that are responsible for maintaining and replenishing the blood and immune systems throughout life.^11,12^ Platelets are derived from megakaryocytes that differentiate from HSCs and downstream progenitors.^13^ HSCs are unique, as they can be replaced through transplantation and therefore serve as an ideal target for one-time therapeutic interventions for a range of blood, immune, and metabolic disorders.^14^ Clinically, HSC transplantation has long been used to treat hematologic malignancies and inherited disorders, and recent advances in gene therapy and gene editing have further expanded its therapeutic reach.^14–17^ For example, the approval of Casgevy (exagamglogene autotemcel) for sickle cell disease and β-thalassemia marks the first clinical translation of targeted HSC engineering by genome editing, establishing a foundation for next-generation therapies.^18,19^ However, despite these advances, the therapeutic application of genome editing in HSCs has largely focused on select monogenic blood and immune disorders, and often applies tools that leave long-term scars in the genome, including undesired on and off-target edits, as well as other heritable gene expression alterations, which limit broader potential applications.^20–23^

Programmable epigenome silencers target specific genomic loci to modulate gene expression without altering the underlying DNA sequence, representing versatile and potentially safer therapeutic modalities to repress disease-linked genes. Leveraging epigenetic memory, the faithful maintenance of cytosine methylation at CpG sites through DNA replication and cell division, systems such as CRISPRoff and CHARM (Coupled Histone tail for Autoinhibition Release of Methyltransferase) establish sustained transcriptional repression in mammalian cells by depositing DNA methylation at targeted loci through exogenous DNMT3A catalytic domains or by recruiting endogenous DNMT3A together with DNMT3L, in addition to incorporating the KRAB repressor domain.^24,25^ These DNA methylation-based silencers have been shown to effectively and durably silence *PCSK9* in the liver to lower circulating cholesterol levels, the prion disease gene *Prnp* in neurons, and specific genes to improve CAR-T persistence.^25–28^ Importantly, such silencing is reversible through targeted DNA demethylation, providing an additional layer of safety for therapeutic applications.^24^ A prior study reported that targeted partial promoter methylation at the *CDKN2B* (p15) locus in human hematopoietic stem and progenitor cells (HSPCs) persisted during myeloid differentiation *in vitro* and in xenotransplanted mice.^29^ However, the comprehensive characterization of targeted epigenome silencing durability during HSC self-renewal and multilineage differentiation, its reversibility, and therapeutic potential in human hematopoiesis remains largely unexplored.

Here, we optimize and apply highly efficient epigenetic silencing in human HSCs. As an initial proof-of-principle target, we chose the high-copy platelet fibrinogen receptor integrin β3 (*ITGB3*), which is required for platelet aggregation and is a key target of antithrombotic therapies.^30–32^ We demonstrate specific and robust *ITGB3* silencing by CHARM that persists through HSC self-renewal and megakaryocytic/hematopoietic differentiation, resulting in a significant reduction of platelet aggregation. Silencing remained stable after long-term engraftment in transplanted mice and serial colony replating. We further demonstrate the reversibility of this approach, as well as the versatility across a range of other antithrombosis targets that function in platelets. These results establish epigenome editing in HSCs as a durable and versatile platform for thrombosis prevention, as well as a potential treatment f for other diseases that have roots in the HSC compartment.

## Results

### Optimization of approaches for programmable epigenetic silencing in human HSCs

**Figure 1:**
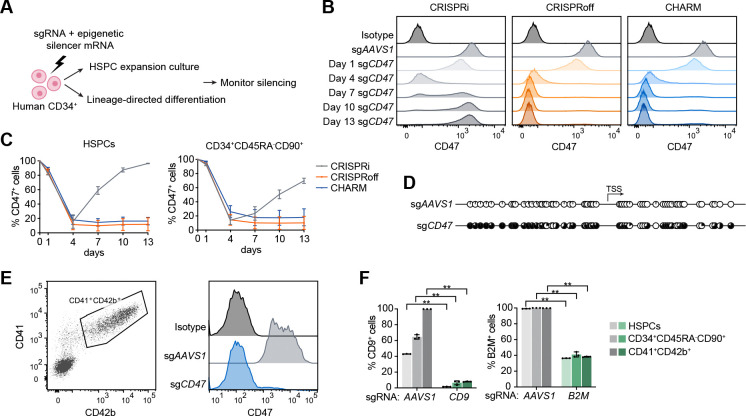
CHARM enables durable epigenetic silencing in human HSPCs. **(A)** Schematic overview of the workflow used to establish epigenetic silencing in primary human CD34^+^ HSPCs. **(B)** Representative flow cytometry histograms of surface CD47 expression over time in HSPCs edited with CRISPRi, CRISPRoff, or CHARM targeting the *CD47* promoter. Isotype staining and *AAVS1*-targeted cells serve as controls. **(C)** Quantification of the percentage of CD47^+^ cells over time in bulk HSPCs (left) and within phenotypically defined CD34^+^CD45RA^−^CD90^+^ HSCs (right) following CRISPRi, CRISPRoff, or CHARM targeting of the CD47 promoter. (D) Targeted bisulfite sequencing displaying CpG methylation patterns near the ITGB3 TSS in HSPCs 13 days after editing with CHARM targeting the CD47 promoter or AAVS1 as a control. Each circle represents an individual CpG site; filled circles indicate methylated CpGs. (E) Left, representative flow cytometry gating strategy used to define CD41^+^CD42b^+^ mature megakaryocytes derived from edited HSPCs. Right, representative flow cytometry histograms of surface CD47 expression in these cells following CHARM targeting of CD47 promoter or AAVS1. (F) Quantification of the percentage of CD9^+^ (left) and B2M^+^ (right) cells across bulk HSPCs and CD34^+^CD45RA^−^CD90^+^-defined HSCs at 13 days after editing, and CD41^+^CD42b^+^ megakaryocytes after 12 days of differentiation, following CHARM targeting in HSPCs. All data are presented as mean ± SD, significance is indicated as *P < 0.05, **P < 0.01, ***P < 0.001, or n.s. not significant.

We first sought to optimize conditions for applying targeted programmable epigenetic silencing in human HSCs and their progeny using RNA delivery (Figure 1A). CD34^+^ HSPCs that were mobilized from healthy adult donors were electroporated with mRNAs encoding CRISPR interference (CRISPRi), CRISPRoff, or CHARM (Figure S1A), together with a single guide RNA (sgRNA) targeting the promoter of *CD47*, selected for its annotated promoter CpG island, cell surface localization, broad hematopoietic expression, and functional neutrality in our *in vitro* culture conditions. Cells were maintained in HSPC expansion culture, and *CD47* expression was monitored by flow cytometry over time. CRISPRi initially abolished *CD47* expression in ~90% of cells, but the effect progressively diminished over several days in culture (Figures 1B-C), consistent with the shortlived nature of RNA delivery and the generally non-heritable characteristics of histone modifications. In contrast, DNA methylation-based silencers, CRISPRoff and CHARM, induced efficient (~80–95% CD47^−^ cells) and persistent silencing in HSPCs throughout an extended culture (Figures 1B-C). Similar repression was observed when the analysis was restricted to the CD34^+^CD45RA^−^CD90^+^ HSC-enriched subset, indicating effective gene silencing in populations enriched for *bona fide* HSCs (Figures 1C and S1B-C). We selected CHARM for subsequent experiments due to its smaller size and favorable safety profile, which align with therapeutic development.^25^ Consistent with an epigenetic mechanism of silencing, CHARM-mediated repression was accompanied by increased DNA methylation surrounding the *CD47* transcription start site (TSS) and concomitantly reduced mRNA expression (Figures 1D and S1D).

To assess whether epigenetic silencing remained stable after differentiation of HSCs, CHARM-edited HSPCs were differentiated toward the megakaryocytic and erythroid lineages. We found that *CD47* repression was stably maintained at the endpoints of both trajectories, with ~95% silencing in CD41^+^CD42b^+^ mature megakaryocytes (MKs) and ~99% silencing in erythroid cultures composed predominantly of intermediate (CD71^+^CD235^+^) and late (CD71^−^CD235^+^) erythroblasts (Figures 1E and S1E-F), indicating preserved repression through lineage commitment and terminal maturation. Importantly, despite known alterations in global DNA methylation during hematopoietic differentiation^33–36^, these results support the stability and effectiveness of this approach.

We then evaluated the generality of CHARM-mediated silencing by targeting two additional surface genes with distinct expression profiles, *CD9* and *B2M*. Both loci exhibited robust and durable repression across HSPCs, HSC-enriched populations, and differentiated MKs, albeit with varying silencing efficiencies (>90% CD9^−^ cells and ~60% B2M^−^ cells) (Figure 1F). Together, these results establish CHARM as an efficient and durable epigenome silencing platform for human HSCs that remains stable upon differentiation.

### Effective and specific *ITGB3* silencing by CHARM

**Figure 2:**
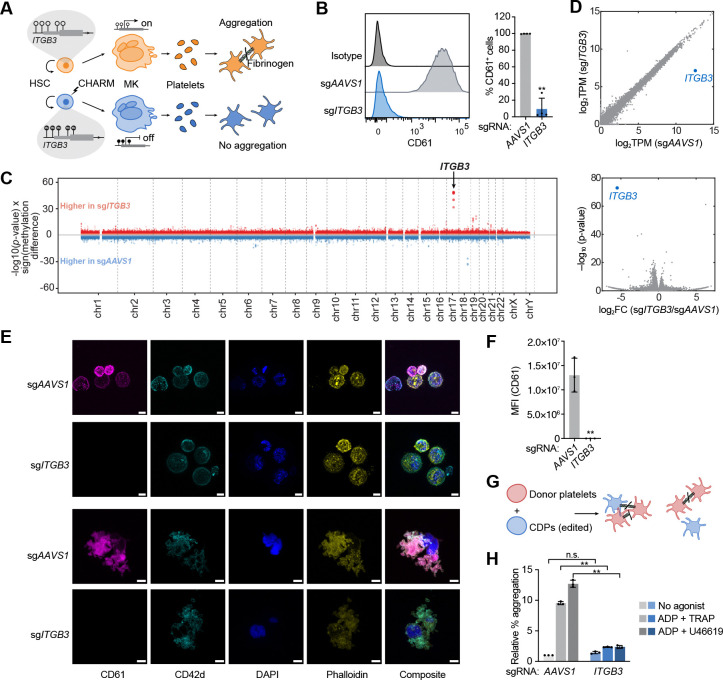
Robust and specific silencing of ITGB3 by CHARM persists through megakaryocytic differentiation and impairs platelet aggregation. (A) Schematic illustrating the strategy for CHARM-mediated silencing of ITGB3 in HSPCs. Targeted methylation established in HSPCs is maintained through self-renewal, megakaryocytic differentiation, and platelet formation, resulting in reduced αIIbβ3 expression and platelet aggregation. (B) Left, representative flow cytometry histograms of CD61 (ITGB3) surface expression in mature megakaryocytes differentiated from HSPCs following CHARM targeting of the ITGB3 promoter or AAVS1. Right, quantification of the percentage of CD61^+^ cells among mature megakaryocytes. (C) Manhattan plot displaying differentially methylated regions between HSPCs edited with CHARM targeting the ITGB3 promoter or AAVS1 as a control, analyzed by WGBS. Red dots indicate CpGs with increased DNA methylation in ITGB3-targeted cells relative to AAVS1 controls, whereas blue dots indicate CpGs with decreased methylation. The arrow denotes the genomic position of ITGB3. (D) RNA sequencing plots of differentiated megakaryocytes edited with CHARM targeting the ITGB3 promoter or AAVS1 as a control. Top, scatter plot showing transcript abundance (log_2_ TPM) for each gene in the two experimental groups, with ITGB3 highlighted in blue. Bottom, volcano plot showing differential gene expression between conditions, with ITGB3 highlighted in blue. (E) Representative immunofluorescence images of megakaryocytes and proplatelets derived from HSPCs edited with CHARM targeting the ITGB3 promoter or AAVS1 as a control. Cells were stained for CD61, CD42d, DAPI, and F-actin (phalloidin). Scale bars, 10 μm. (F) Quantification of CD61 MFI measured by IF imaging in 20–30 individual megakaryocytes identified by CD42d staining per condition. (G) Schematic of the platelet aggregation assay assessing the ability of CDPs generated from edited HSPCs to aggregate with donor platelets. (H) Quantification of relative platelet aggregation in CDPs generated from HSPCs edited with CHARM targeting the ITGB3 promoter or AAVS1 as a control, measured as the percentage of double-positive platelet-CDP events normalized to total CDP events under no-agonist conditions or following stimulation with ADP + TRAP or ADP + U46619. All data are presented as mean ± SD, significance is indicated as *P < 0.05, **P < 0.01, ***P < 0.001, or n.s. not significant.

The persistence of gene silencing in human HSCs and their differentiated progeny would enable long-term therapeutic benefit. We reasoned that this property could be leveraged to silence genes essential for platelet adhesion, activation, and aggregation, thus serving to prevent thrombosis. A key target to prevent thrombosis is the integrin αIIbβ3 (encoded by *ITGA2B* and *ITGB3*), a central mediator of platelet aggregation that acts through fibrinogen binding.^30,31^ Loss-of-function variants in *ITGA2B* and *ITGB3* cause Glanzmann thrombasthenia, a bleeding disorder that lacks other phenotypic manifestations; clinically, αIIbβ3 inhibitors are used to prevent thrombotic complications, particularly during acute interventions.^37,38^ Extensive prior work has defined the central role of *ITGB3* in platelet aggregation, and its CpG-rich promoter architecture makes it particularly well suited for CHARM-mediated methylation, which collectively nominate *ITGB3* an ideal proof-of-principle target.^30–32,39^ We hypothesized that CHARM-induced methylation at the *ITGB3* promoter would persist through HSC self-renewal and megakaryocytic differentiation, leading to stable silencing in MKs and derived platelets and, consequently, impaired aggregation (Figure 2A).

In this context, we first examined whether *ITGB3* could be silenced by CHARM in HSPCs and maintained through megakaryocytic differentiation. CD34^+^ HSPCs were electroporated with CHARM mRNA together with an sgRNA targeting either the *AAVS1* control locus or the *ITGB3* promoter. Following differentiation, the latter induced robust silencing of *ITGB3*, with an average of ~90% CD61 (encoded by *ITGB3*) negative cells among mature MKs (Figure 2B). In line with promoter-targeted epigenetic repression, we observed a significant increase in DNA methylation near the *ITGB3* TSS and a reduction in *ITGB3* mRNA expression (Figures S2B-C).

To assess the specificity of CHARM-mediated DNA methylation, we performed whole-genome bisulfite sequencing (WGBS) on modified HSPCs. Compared with *AAVS1* targeting controls, the most prominent increase in DNA methylation was observed at the targeted *ITGB3* locus on chromosome 17 (Figure 2C). Consistent with prior reports, in addition to focal methylation at the sgRNA targeting site, DNA methylation spread across the CpG-rich region surrounding the *ITGB3* promoter (~1.5 kb) (Figure S2D). Off-target differentially methylated regions (DMRs) identified in HSPCs were subsequently hierarchically annotated based on genomic context (Figure S2E). Notably, *in silico*-predicted sgRNA off-target sites did not overlap these regions. Although seven off-target DMRs were annotated as promoter-associated, inspection of these loci revealed that differential methylation tended to involve limited regions and rarely overlapped core promoter CpG islands or TSSs (Figure S2F). To further examine transcriptional consequences, RNA sequencing was performed on differentiated and sorted CD42b^+^ MKs. We found that gene repression was highly specific to *ITGB3*, while other transcripts, including those associated with offtarget DMRs identified by WGBS, remained largely unchanged. The only notable exception was up-regulation of the neighboring gene of *ITGB3*, *MYL4*, likely reflecting local chromatin remodeling at the silenced locus, as has been observed with DNA methylation changes occurring during hematopoietic differentiation (Figure 2D).^36^

### Disruption of platelet aggregation by CHARM-mediated *ITGB3* silencing

To further assess *ITGB3* silencing during MK maturation and proplatelet formation, we visualized the cells through immunofluorescence imaging. We observed mature, round, CD42-positive, polyploid MKs and readily detected proplatelet-forming structures, confirming the presence of morphologically conventional MKs. While control cells exhibited clear CD61 labeling, CHARM *ITGB3*-edited cells showed near-complete loss of CD61 signal, indicating efficient suppression of *ITGB3* expression (Figures 2E-F).

We next sought to directly test whether *ITGB3* silencing in HSPCs leads to functional impairment in the aggregation of derived platelets. Although culture-derived platelets (CDPs) produced *in vitro* from human MKs have reduced function compared to platelets produced *in vivo*, they can be valuable surrogates to examine physiological responses when mixed with blood-derived platelets.^40–43^ We therefore employed a flow cytometry-based assay to assess the ability of CDPs generated from edited cells^43^ (Figure S2G) to aggregate with healthy donor platelets (Figure 2G). Donor platelets and CDPs were differentially labeled, mixed, and stimulated with agonists before fixation and flow cytometry (Figure S2H). Double-positive events, representing platelet-CDP aggregates, were quantified as a measure of CDP aggregation capacity. While control CDPs exhibit robust aggregation upon stimulation with ADP together with the PAR1 agonist peptide TRAP or the thromboxane A_2_ analog U46619, those derived from *ITGB3*-silenced cells displayed a pronounced defect in forming aggregates with donor platelets, consistent with the essential role of αIIbβ3 in platelet-platelet interactions that are induced by a variety of platelet activators (Figures 2H and S2I). Collectively, these results demonstrate that silencing of *ITGB3* by CHARM persists through MK differentiation, generating platelets that lack this key fibrinogen receptor, thereby impairing platelet aggregation.

### Persistence of *ITGB3* silencing by CHARM through HSC self-renewal

**Figure 3:**
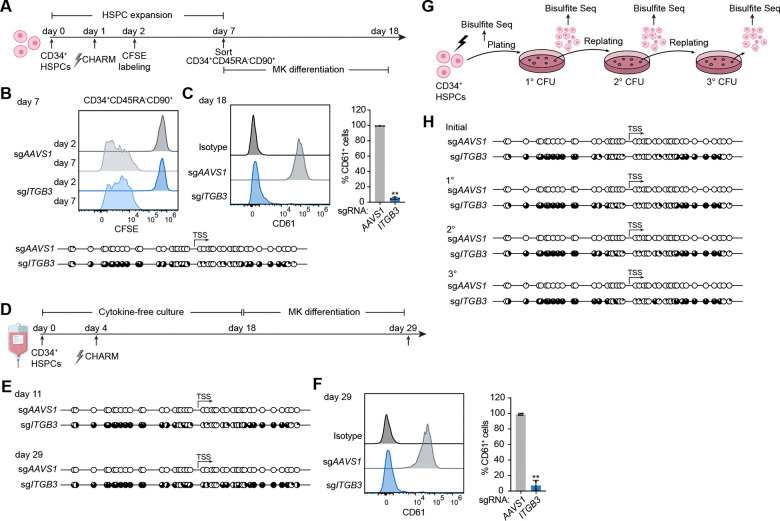
CHARM-mediated *ITGB3* silencing persists through HSC self-renewal. **(A)** Schematic overview of the experimental design for CHARM editing, tracking and sorting of CD34^+^CD45RA^−^CD90^+^ cells, and subsequent megakaryocytic differentiation. **(B)** Top, representative CFSE dilution profiles of CD34^+^CD45RA^−^CD90^+^ cells at days 2 and 7 following CHARM targeting of the *ITGB3* promoter or *AAVS1* as a control. Bottom, targeted bisulfite sequencing displaying CpG methylation patterns near the *ITGB3* TSS in the sorted CD34^+^CD45RA^−^CD90^+^ cells. Each circle represents an individual CpG site; filled circles indicate methylated CpGs. **(C)** Left, representative flow cytometry histograms of CD61 (*ITGB3*) surface expression in megakaryocytes differentiated from sorted CD34^+^CD45RA^−^CD90^+^ cells following CHARM targeting of the *ITGB3* promoter or *AAVS1* as a control. Right, quantification of the percentage of CD61^+^ cells among differentiated megakaryocytes. **(D)** Schematic overview of the experimental design for extended cytokine-free culture and subsequent megakaryocytic differentiation following CHARM editing of HSPCs. **(E)** Targeted bisulfite sequencing displaying CpG methylation patterns near the *ITGB3* TSS in HSPCs at day 11 and day 29 following CHARM targeting of the *ITGB3* promoter or AAVS1 as a control. Each circle represents an individual CpG site; filled circles indicate methylated CpGs. **(F)** Left, representative flow cytometry histograms of CD61 (*ITGB3*) surface expression in megakaryocytes differentiated from HSPCs after extended cytokine-free culture following CHARM targeting of the *ITGB3* promoter or *AAVS1* as a control. Right, quantification of the percentage of CD61^+^ cells among differentiated megakaryocytes. **(G)** Schematic overview of the experimental design for serial CFU replating and targeted bisulfite sequencing following CHARM editing of HSPCs. **(H)** Targeted bisulfite sequencing displaying CpG methylation patterns near the *ITGB3* TSS in HSPCs after CHARM editing and in cells derived from primary (1°), secondary (2°), and tertiary (3°) CFU replatings following targeting of the *ITGB3* promoter or AAVS1 as a control. Each circle represents an individual CpG site; filled circles indicate methylated CpGs. All data are presented as mean ± SD, significance is indicated as *P < 0.05, **P < 0.01, ***P < 0.001, or n.s. not significant.

Beyond differentiation, effective HSC-based therapies require that therapeutic editing outcomes be maintained as HSCs undergo self-renewal throughout the lifespan. We therefore sought to rigorously examine persistence of silencing during HSC self-renewal and long-term culture. To track division, CHARM-edited HSPCs were labeled with carboxyfluorescein succinimidyl ester (CFSE). Division of phenotypically defined CD34^+^CD45RA^−^CD90^+^ HSC-enriched populations was observed over five days of expansion culture (Figure 3A). We found that DNA methylation surrounding the *ITGB3* TSS was well preserved in these cells, indicating maintenance of targeted DNA methylation through division of primitive HSPCs (Figure 3B). These sorted HSCs were further subjected to megakaryocytic differentiation, during which *ITGB3* silencing was robustly retained in mature MKs, as assessed by a lack of CD61 expression, demonstrating transmission of silencing from dividing HSCs to their differentiated progeny (Figure 3C). Consistent with the specificity of editing, apoptosis, cell viability, and the maintenance of phenotypically defined short- and long-term repopulating (ST/LT) HSCs were not affected (Figures S3A-B).

To enable longer-term evaluation of silencing stability, edited cells were maintained in a chemically defined, cytokine-free culture which maximizes HSC self-renewal *in vitro* for three weeks prior to megakaryocytic differentiation (Figure 3D).^44,45^ Throughout this extended culture, we found that DNA methylation at the *ITGB3* TSS remained stable (Figure 3E). At the endpoint of differentiation, loss of CD61 expression was still observed in mature MKs, indicating that *ITGB3* silencing was maintained after prolonged culture (Figure 3F)

Lastly, to assess maintenance of DNA methylation during functional HSPC self-renewal and multilineage differentiation, we performed serial replating assays. Edited HSPCs were plated in methylcellulose, and colonies were harvested and replated sequentially to generate secondary and tertiary colonies that depend upon progenitor and stem cell self-renewal (Figures 3G and S3C). Targeted DNA methylation near the *ITGB3* TSS was examined after each replating, and was faithfully maintained through tertiary replating (Figure 3H). These results indicate stable transmission of CHARM-induced DNA methylation across repeated cycles of HSPC self-renewal and differentiation.

### Sustained silencing during long-term engraftment in mice

**Figure 4:**
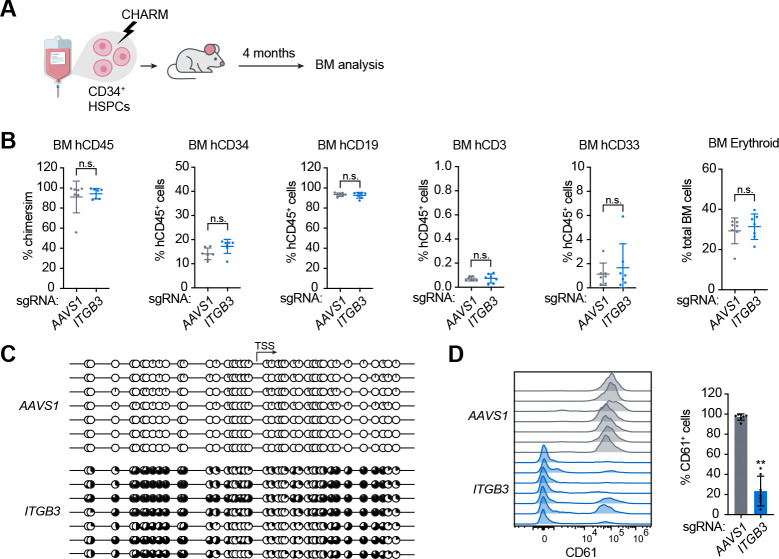
*ITGB3* silencing by CHARM is maintained following long-term engraftment in xenotransplanted mice. **(A)** Schematic overview of xenotransplantation of CHARM-edited human CD34^+^ HSPCs into NBSGW mice and subsequent analysis 16 weeks after transplantation. (B) Quantification of human hematopoietic engraftment in the bone marrow of recipient mice 16 weeks after transplantation, assessed by overall chimerism (% hCD45^+^ cells), and frequency of human CD34^+^ compartment, B (hCD19^+^), T (hCD3^+^), myeloid (hCD33^+^), and erythroid populations. (C) Targeted bisulfite sequencing displaying CpG methylation patterns near the *ITGB3* TSS in human CD34^+^ cells isolated from transplanted mice 16 weeks after engraftment. Each circle represents an individual CpG site; filled circles indicate CpGs with high DNA methylation. (D) Representative flow cytometry histograms of CD61 (*ITGB3*) surface expression in megakaryocytes differentiated from human CD34^+^ cells recovered from mice transplanted with CHARM-edited HSPCs targeting the *ITGB3* promoter or *AAVS1* as a control. All data are presented as mean ± SD, significance is indicated as *P < 0.05, **P < 0.01, ***P < 0.001, or n.s. not significant.

To further assess the long-term persistence and functional output of edited human HSCs *in vivo*, we performed xenotransplantation of edited human CD34^+^ HSPCs into *Kit* mutant and immunodeficient NOD.Cg-*Kit*^*W−41J*^*Tyr*^+^*Prkdc*^*scid*^*Il2rg*^*tm1Wjl*^/ThomJ (NBSGW) mice (Figure 4A). 4 months post transplantation, after long-term engraftment by human HSCs is well established, bone marrow was harvested for analysis.

Robust and comparable human hematopoietic engraftment was observed in recipient mice transplanted with CHARM-edited HSPCs targeting either the *AAVS1* locus or the *ITGB3* promoter, as assessed by the frequency of hCD45^+^ cells in the bone marrow (Figure 4B). Similarly, the contribution of the CD34^+^ compartment and major hematopoietic lineages, including B (CD19^+^), T (CD3^+^), myeloid (CD33^+^), and erythroid populations, was comparable between the two groups (Figures 4B and S4A-C), demonstrating a lack of undesired effects on HSPC engraftment or lineage output.

To examine whether CHARM-mediated epigenetic modification at the *ITGB3* locus was maintained in engrafting human progenitors after self-renewal of HSCs post-transplant and residence in the bone marrow niche, we performed bisulfite sequencing on human CD34^+^ cells isolated from the bone marrows of transplanted mice. Sustained DNA methylation near the *ITGB3* TSS was observed in all mice in the *ITGB3* targeting group (Figure 4C). Given that few human platelets or megakaryocytes were generated in these mice, we subjected CD34^+^ cells from long-term engrafted mice to megakaryocytic differentiation, and found that *ITGB3* silencing was robustly retained in mature MKs, with on average ~80% of cells lacking CD61 expression (Figure 4D).

### Reversibility of silencing through targeted demethylation

**Figure 5:**
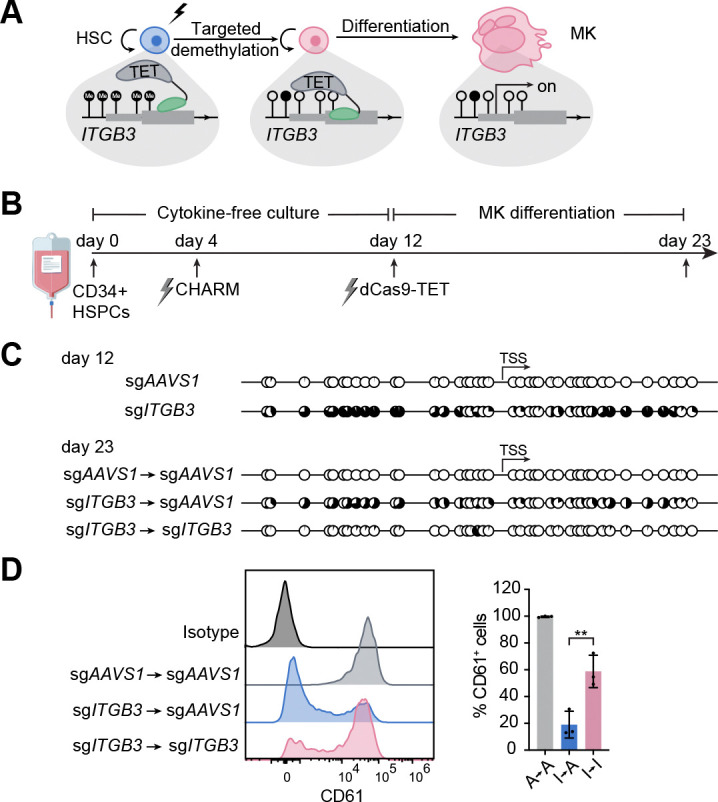
Targeted demethylation reverses CHARM-mediated *ITGB3* silencing. **(A)** Schematic illustrating targeted demethylation of the *ITGB3* promoter in HSCs using dCas9-TET following CHARM-mediated silencing, and subsequent megakaryocytic differentiation. **(B)** Schematic overview of the experimental design for sequential CHARM-mediated methylation and dCas9-TET-mediated demethylation in HSPCs, followed by megakaryocytic differentiation. **(C)** Targeted bisulfite sequencing displaying CpG methylation patterns near the *ITGB3* TSS in HSPCs at day 12 (before targeted demethylation) and day 23 (after targeted demethylation and megakaryocytic differentiation), following CHARM and dCas9-TET targeting as indicated. Each circle represents an individual CpG site; filled circles indicate methylated CpGs. **(D)** Left, representative flow cytometry histograms of CD61 (*ITGB3*) surface expression in megakaryocytes differentiated from HSPCs subjected to sequential CHARM and dCas9-TET targeting as indicated. Right, quantification of the percentage of CD61^+^ cells among megakaryocytes. All data are presented as mean ± SD, significance is indicated as *P < 0.05, **P < 0.01, ***P < 0.001, or n.s. not significant.

One key feature of epigenome editing is its reversibility, enabling restoration of gene expression if required in a therapeutic context.^24,27^ We therefore tested whether targeted demethylation using TET1-dCas9 could reverse CHARM-mediated repression by erasing methylation marks at the silenced promoter (Figure 5A). To this end, CHARM-edited HSPCs were maintained in cytokine-free culture for eight days and then a second electroporation was performed with mRNA encoding TET1-dCas9, together with the sgRNAs targeting either the *AAVS1* control locus or the *ITGB3* promoter (Figure 5B). Following megakaryocytic differentiation, the latter induced significant demethylation near the *ITGB3* TSS (Figure 5C). Correspondingly, surface CD61 expression was restored (Figure 5D). These results demonstrate that targeted demethylation enables controlled reversal of silencing in human HSPCs.

### Broad applicability of CHARM in platelet targets

**Figure 6:**
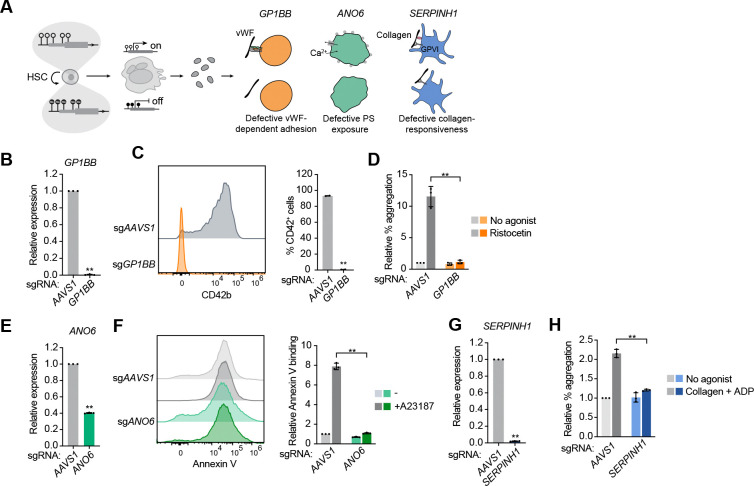
CHARM is broadly applicable for epigenetic silencing of multiple platelet genes in HSPCs. **(A)** Schematic illustrating CHARMmediated epigenetic silencing in HSPCs and its application to distinct platelet genes acting at different stages of thrombus formation, including *GP1BB*, *ANO6*, and *SERPINH1*. **(B)** Quantification of *GP1BB* mRNA expression measured by RT-qPCR in differentiated megakaryocytes following CHARM targeting of the *GP1BB* promoter or *AAVS1* as a control. **(C)** Left, representative flow cytometry histograms of CD42b surface expression in CD41^+^GP6^+^ megakaryocytes differentiated from HSPCs following CHARM targeting of the *GP1BB* promoter or *AAVS1*. Right, quantification of the percentage of CD42b^+^ cells among megakaryocytes. **(D)** Quantification of relative platelet aggregation in CDPs generated from HSPCs edited with CHARM targeting the *GP1BB* promoter or *AAVS1* as a control, measured as the percentage of double-positive platelet-CDP events normalized to total CDP events under no-agonist conditions or following stimulation with ristocetin. **(E)** Quantification of *ANO6* mRNA expression measured by RT-qPCR in differentiated CD41^+^CD42b^+^ megakaryocytes following CHARM targeting of the *ANO6* promoter or *AAVS1* as a control. **(F)** Left, representative flow cytometry histograms of Annexin V binding in CDPs generated from HSPCs edited with CHARM targeting the *ANO6* promoter or *AAVS1* under baseline conditions or following stimulation with the Ca²^+^ ionophore A23187. Right, quantification of relative Annexin V binding. **(G)** Quantification of *SERPINH1* mRNA expression measured by RT-qPCR in differentiated CD41^+^CD42b^+^ megakaryocytes following CHARM targeting of the *SERPINH1* promoter or *AAVS1* as a control. **(H)** Quantification of relative platelet aggregation in CDPs generated from HSPCs edited with CHARM targeting the *SERPINH1* promoter or *AAVS1* as a control, measured as the percentage of double-positive platelet-CDP events normalized to total CDP events under no-agonist conditions or following stimulation with collagen + ADP. All data are presented as mean ± SD, significance is indicated as *P < 0.05, **P < 0.01, ***P < 0.001, or n.s. not significant.

While the integrin αIIbβ3 represents a valuable initial proof-of-principle target to demonstrate the effectiveness of CHARM to prevent platelet aggregation after HSC editing, therapeutic modulation may benefit from consideration of additional platelet targets acting at different steps of thrombus formation, which would also have differing risks of bleeding and thereby provide variable safety profiles. We therefore asked whether CHARM-mediated silencing in HSPCs could be broadly applied to additional platelet genes involved in thrombus formation, which also have human genetic validation, as alternative targets for thrombosis prevention (Figure 6A).

We first targeted *GP1BB*, which encodes a component of the GPIb-IX-V complex that mediates von Willebrand factor (vWF)-dependent platelet adhesion under high shear conditions.^46^ This target has been validated through loss-of-function human genetic mutations in the gene that result in the bleeding disorder Bernard-Soulier syndrome.^47^ CHARM targeting of the *GP1BB* promoter in HSPCs resulted in increased DNA methylation at the targeted locus in differentiated MKs (Figure S5A), accompanied by near-complete repression of *GP1BB* mRNA expression (Figure 6B). This silencing led to loss of surface CD42b (GPIbα), consistent with destabilization of the GPIb complex (Figure 6C). Functionally, CDPs generated from *GP1BB*-silenced HSPCs exhibited a profound defect in ristocetin-induced aggregation with donor platelets, demonstrating impaired GPIb-vWF-dependent platelet aggregation (Figure 6D and S5B).

We next tested CHARM-mediated silencing of *ANO6*, which encodes a Ca²^+^-activated phospholipid scramblase that is essential for platelet procoagulant activity through phosphatidylserine (PS) exposure.^48–50^ Loss-of-function mutations in this gene cause Scott syndrome, a mild bleeding disorder.^48^ Editing of *ANO6* by CHARM led to a partial but substantial reduction (~60%) in *ANO6* mRNA expression in differentiated MKs, with corresponding increased DNA methylation near the TSS (Figure 6E and Figure S5C). At baseline, CDPs displayed some Annexin V binding (indicating surface PS exposure), consistent with partial membrane remodeling and pre-activation commonly observed in platelets derived from culture. However, stimulation with the Ca²^+^ ionophore A23187 induced a pronounced loss of the PS-negative population in control CDPs, whereas this response was markedly attenuated in *ANO6*-silenced CDPs (Figure 6F), indicating impaired Ca²^+^-triggered PS externalization.

Finally, we examined the potential of silencing *SERPINH1* (HSP47), a chaperone implicated in platelet activation downstream of collagen engagement.^51,52^ Interestingly, reduced *SERPINH1* expression in platelets has been suggested to prevent thrombosis in hibernating animals, such as bears, and in immobile humans.^51^ *SERPINH1* targeting by CHARM in HSPCs led to robust DNA methylation near the TSS and near-complete loss of *SERPINH1* mRNA expression in differentiated MKs (Figure 6G and Figure S5D). Although collagen-induced aggregation was modest in our assay, CDPs generated from *SERPINH1*-silenced cells exhibited a significantly reduced response relative to controls (Figure 6H and Figure S5E).

## Discussion

Advances in HSC-based therapies have enabled one-time curative treatment of hematologic malignancies and inherited disorders; however, their broader potential remains underexploited given the diversity of HSC-derived cell types and their systemic roles. One such opportunity is thrombosis, a common and clinically important condition for which effective therapies exist, but which typically require lifelong administration and can show variable effectiveness due to persistently elevated platelet reactivity. Thus, modulation of platelet genes essential for thrombus formation in HSCs is an attractive strategy. Compared with genome editing, epigenome editing modulates gene expression without altering the underlying DNA sequence and retains the capacity for reversibility, offering a potentially safer therapeutic modality. Within this class of approaches, DNA methylation-based silencers can achieve durable repression through deposition of heritable methylation marks following transient delivery. Such strategies have been successfully applied to silence disease-causing genes in post-mitotic tissues such as the brain, and in the liver, where cells undergo limited proliferation.^25–27^ In contrast, the understudied HSCs represent a particularly demanding context, as methylation and silencing must be faithfully propagated through extensive cell division that both sustains self-renewal and generates differentiated hematopoietic progeny. Here, by targeting the platelet receptor integrin β3 (*ITGB3*), a central mediator of platelet aggregation, we show that its silencing by CHARM in human HSPCs can be durably inherited through HSC self-renewal and megakaryocytic differentiation, with functional consequences for platelet aggregation.

From a safety perspective, CHARM-mediated editing of *ITGB3* was highly specific. Methylome and transcriptome profiling revealed minimal off-target effects on DNA methylation and gene expression. At the targeted locus, we observed localized transcriptional changes, including notable up-regulation of the neighboring gene *MYL4*, likely due to local chromatin remodeling; however, this on-target effect did not elicit secondary alterations in global transcriptional programs. Consistent with this specificity, CHARM editing did not affect HSC viability, apoptosis, short- or long-term HSC maintenance, colony-forming potential *in vitro*, or engraftment and multilineage reconstitution *in vivo*. Importantly, CHARM-mediated silencing is reversible through targeted demethylation, highlighting a key control feature of epigenome editing in human HSCs.

Of note, we used *ITGB3* as a proof-of-principle target based on its CpG-rich promoter, well-defined role in platelet aggregation, favorable safety profile inferred from human genetics, and clinical precedent demonstrating that inhibition of αIIbβ3 reduces thrombotic events.^32,38,39,53^ Extending beyond this single target, we demonstrated the broader applicability of CHARM-mediated silencing to genes acting at different steps of thrombus formation and thus with distinct effectiveness-risk profiles. Together, our study establishes epigenome editing of human HSCs as a new therapeutic paradigm for thrombosis prevention. More broadly, it defines HSC-based epigenome editing as a general approach for sustained and controllable regulation of hematopoietic gene expression across diseases. While this study did not focus on delivery, we envision that the engineering of HSCs could be applied *ex vivo*, as current therapies are delivered, or *in vivo*, as emerging approaches for HSC delivery such as lipid nanoparticles and virus-like particles continue to rapidly advance.^54–56^

### Limitations of the study

While the NBSGW xenotransplantation model is optimized for supporting long-term human hematopoiesis, human megakaryopoiesis and platelet production only occur at low levels in this system, and circulating human platelets are extremely rare. As a result, *in vivo* functional assessment of platelet activity and thrombus formation following epigenome editing was not feasible in this model. In addition, DNA methylation-based epigenome silencing would only be effective at loci containing CpG-rich regulatory regions with low baseline methylation. Accordingly, in this study we focused on genetically-inspired antiplatelet targets with annotated CpG islands at their promoters. We did not systematically evaluate genes lacking promoter CpG islands, nor did we explore targeting of distal regulatory elements such as enhancers, which may expand the range of genes amenable to epigenetic modulation.

## Materials and Methods

### Primary cell culture

All experiments were conducted in accordance with institutional guidelines and approval at Boston Children’s Hospital.

CD34^+^ HSPCs isolated from G-CSF-mobilized peripheral blood of adult donors were purchased from the Fred Hutchinson Cancer Research Center. Cryopreserved cells were thawed rapidly in a 37 °C water bath and gradually diluted with thawing medium (DPBS + 1% FBS). Cells were then cultured at 37 °C in StemSpan Serum-Free Expansion Medium (SFEM) II (STEMCELL Technologies) supplemented with L-glutamine (2 mM), penicillin-streptomycin (100 U/mL), StemSpan CC100 (1×; STEMCELL Technologies), thrombopoietin (TPO) (50 ng/mL; PeproTech), and UM171 (35 nM; STEMCELL Technologies).

Cord blood was obtained from the Brigham and Women’s Hospital Center for Clinical Investigation. CD34^+^ cells were enriched using the EasySep Human CD34 Positive Selection Kit (STEMCELL Technologies) according to the manufacturer’s instructions and were either cryopreserved until use or directly cultured.

For chemically defined cytokine-free culture, cells were cultured in Iscove’s modified Dulbecco’s medium (IMDM; Gibco) supplemented with 1% insulin-transferrin-selenium-ethanolamine (Life Technologies), penicillin-streptomycin (100 U/mL), UM171 (70 nM), polyvinyl alcohol (1 mg/mL; Sigma-Aldrich), 740Y-P (1 μM; MedChemExpress), and butyzamide (0.1 μM; MedChemExpress). Cells were maintained at a density of 5 × 10^4^ to 1 × 10^5^ cells per mL in CellBIND plates (Corning).

For megakaryocytic differentiation, edited cells were cultured in StemSpan Serum-Free Expansion Medium (SFEM) II supplemented with penicillin-streptomycin (100 U/mL), human stem cell factor (SCF; 100 ng/mL), TPO (100 ng/mL), Flt-3 ligand (100 ng/mL; PeproTech), and IL-6 (100 ng/mL; PeproTech) for 6 days, followed by culture in StemSpan SFEM II supplemented with penicillin-streptomycin (100 U/mL) and TPO (50 ng/mL) for an additional 6 days. For proplatelet formation experiments, heparin (25 U/mL) was added to the latter differentiation medium. In experiments generating CDPs, SR1 (1 μM; MedChemExpress) and human low-density lipoprotein (LDL; 20 μg/mL; Sigma-Aldrich) were added to both media.

For erythroid differentiation, a previously described three-phase culture system was used.^57^ The base erythroid medium consisted of IMDM supplemented with 2% human AB plasma (SeraCare), 3% human AB serum (Thermo Fisher Scientific), heparin (3 U/mL), insulin (10 μg/mL), holotransferrin (200 μg/mL), and penicillin-streptomycin (100 U/mL). During days 1–7, the base medium was further supplemented with erythropoietin (EPO; 3 U/mL), human SCF (10 ng/mL), and IL-3 (1 ng/mL). From days 8-12, cultures were maintained in base medium supplemented with EPO (3 U/mL) and human SCF (10 ng/mL). From days 13–20, cells were cultured in base medium supplemented with EPO (3 U/mL) and holo-transferrin at a final concentration of 1 mg/mL.

### Mouse models

All mouse procedures were conducted in accordance with protocols approved by the Institutional Animal Care and Use Committee at Boston Children’s Hospital.

CD34^+^ cells enriched from cord blood were nucleofected with CHARM mRNA together with sgRNA targeting either the *AAVS1* locus (control) or the *ITGB3* promoter. Two days after nucleofection, 2 × 10^5^ CD34^+^ HSPCs were intravenously injected via the tail vein into NBSGW mice. Sixteen weeks after transplantation, mice were euthanized by CO_2_ inhalation, and total bone marrow cells were harvested from the ilia, femurs, and tibias. A fraction (3%) of the bone marrow was reserved for erythroid staining, while the remaining cells were subjected to red blood cell lysis followed by engraftment flow cytometric analysis, CD34^+^ cell enrichment, and downstream analyses. Both male and female mice were used for all experiments.

### mRNA production

Coding regions of CRISPRi, CRISPRoff, CHARM, and TET1-dCas9 were cloned into the PEmaxNG-_2star_IVT (Addgene) backbone, which contains sequences of a T7 promoter and optimized 5′ and 3′ untranslated regions (UTRs). Full-length DNA templates were generated by PCR amplification using a reverse primer containing a 5′ poly(T) tract to add a poly(A) tail. PCR products were purified using the Monarch PCR & DNA Cleanup Kit (NEB), and *in vitro* transcription was performed following the Standard-Yield Protocol associated with CleanCap Reagent M6 (TriLink), with reactions incubated at 37 °C for 3 h. Template DNA was removed by treatment with DNase I in the presence of 10× DNase buffer (NEB) at 37 °C for an additional 15 minutes. RNA was purified using the Monarch Spin RNA Cleanup Kit (NEB), quantified by spectrophotometry, and stored at −80 °C until use.

### Editing of CD34^+^ HSPCs

Electroporation of human CD34^+^ HSPCs was performed using the Lonza 4D-Nucleofector system with the P3 Primary Cell 4D-Nucleofector kit (20 μL format). Briefly, cells were harvested, washed twice with DPBS, and resuspended in P3 Primary Cell Nucleofector Solution supplemented with Supplement 1. Cell suspensions were mixed with 100 pmol chemically modified sgRNAs (Synthego) and 2 μg of purified mRNA, transferred to cuvettes, and nucleofected using program DS130.

### Cell staining and flow cytometric analysis

For surface staining, cells were harvested and washed with FACS buffer (DPBS + 2% FBS + 2 mM EDTA), then resuspended in a panel of fluorophore-conjugated antibodies diluted in FACS buffer. Samples were stained at room temperature for 30 min protected from light, washed, resuspended in FACS buffer, and analyzed on a BD LSRFortessa or Cytek Aurora flow cytometer. Data were analyzed using FlowJo.

For division tracing, one day after nucleofection, cells were labeled with CellTrace CFSE (Thermo Fisher) according to the manufacturer’s instructions. Briefly, cells were harvested, washed twice with DPBS, and resuspended in 0.5 μM CFSE diluted in DPBS. After incubation at 37 °C for 10 minutes protected from light, labeling was quenched by the addition of five volumes of FACS buffer. Cells were then washed twice with DPBS and returned to culture. CFSE fluorescence was assessed by flow cytometric analysis on a Cytek Aurora flow cytometer at day 0 and day 5 after labeling. Data were analyzed using FlowJo.

For apoptosis/viability analysis, cells were harvested and washed once with Annexin V binding buffer (BioLegend), then resuspended in Annexin V binding buffer containing Annexin V and DAPI. Samples were incubated for 15 min at room temperature protected from light, and analyzed on a Cytek Aurora flow cytometer. Data were analyzed using FlowJo.

### Colony formation assay

Colony formation assays were performed using MethoCult H4034 Optimum (STEMCELL Technologies) according to the manufacturer’s instructions. Specifically, two days after electroporation, cells were counted, mixed with MethoCult medium, and evenly dispensed into SmartDish (STEMCELL Technologies), with 500 cells plated per well. Colonies were imaged after 12–15 days and harvested by washing with cold DPBS. For serial replating assays, secondary and tertiary colony formation was performed under the same conditions, except that 50,000 and 300,000 cells were used for both secondary and tertiary platings, respectively.

### Immunofluorescence (IF) imaging of megakaryocyte and proplatelets

At the end of megakaryocytic differentiation, cells were harvested and size-selected using a 20 μm cell strainer. The retained fraction was fixed with 4% paraformaldehyde diluted in DPBS at room temperature for 30 min, washed once with DPBS, and blocked in 3% BSA diluted in DPBS at room temperature for 30 min. Cells were then stained with the indicated antibodies overnight at 4 °C. After washing, cells were resuspended in DPBS and imaged.

For proplatelet formation and imaging, Lab-Tek chamber slides were coated with anti-CD31 antibody at 37 °C for 1 h and blocked with 3% BSA diluted in PBS. Cells were harvested, size-selected using a 20 μm cell strainer, and seeded onto the coated chambers. Cells were incubated overnight at 37 °C to allow proplatelet formation. Culture medium was then gently removed, and cells were fixed with 4% paraformaldehyde diluted in DPBS at room temperature for 30 min, washed once with DPBS, and blocked with 3% BSA diluted in DPBS at room temperature for 30 min. Cells were incubated with the indicated antibodies overnight at 4 °C. After removal of the antibody solution, cells were maintained in DPBS and imaged.

Fluorescence Z-stacks were processed in Fiji and displayed as maximum intensity projections; sg*AAVS1* and sg*ITGB3* samples were imaged using identical acquisition settings, and brightness and contrast were adjusted for visualization.

### Targeted bisulfite sequencing

Genomic DNA (gDNA) was extracted from cultured or sorted cells using Quick-DNA Microprep Plus Kit or Quick-DNA/RNA Microprep Plus Kit (Zymo). For each sample, 200–1000 ng of gDNA was subjected to bisulfite conversion using the EZ DNA Methylation-Lightning Kit (Zymo) following the manufacturer’s instructions. Targeted regions were PCR-amplified using primers designed with Bisulfite Primer Seeker (Zymo) and Q5U Hot Start High-Fidelity DNA Polymerase (NEB). Amplicons were sequenced, and raw FASTQ files were filtered by read length and analyzed using QUMA to quantify CpG methylation status.

### qPCR

RNA was isolated from cultured or flow-sorted cells using the Quick-RNA Microprep Plus Kit (Zymo). Equal amounts of RNA (up to 1 μg) were subjected to reverse transcription using PrimeScript RT Master Mix (TaKaRa). Quantitative PCR was performed using primers specifically targeting the indicated genes and SYBR Green Universal Master Mix (Thermo Fisher Scientific). Relative gene expression levels were normalized to *GAPDH* and calculated using the ΔΔCt method, with control samples serving as the reference.

### RNA sequencing

RNA was extracted from CHARM-edited, differentiated, and sorted MKs using the Total RNA Purification Micro Kit (Norgen Biotek) according to the manufacturer’s instructions. Samples were processed and sequenced using Genewiz RNA-seq service. All RNA-seq data were aligned to the human reference genome (hg38) using STAR^58^ with the parameter --runMode alignReads --chimSegmentMin 20. The resulting alignments were sorted, and unmapped reads were removed using SAMtools^59^ with the option -F 1804. Gene-level read counts were then quantified using featureCounts^60^ based on the human reference gene annotation. Raw read counts were normalized to Transcripts Per Million (TPM) to account for both sequencing depth and gene length. For each gene, read counts were first adjusted by gene length to obtain expression values in reads per kilobase. These values were then scaled by the total expression of all genes in the same sample and multiplied by one million to generate TPM values, ensuring that the sum of TPM values within each sample equals one million and allowing direct comparison of gene expression levels across samples.

### Whole-genome bisulfite sequencing

gDNA was extracted from CHARM-edited HSPCs using Quick-DNA Microprep Plus Kit (ZYMO) 4 days post electroporation. Samples were processed and sequenced using Genewiz WGBS service. Raw WGBS-seq FASTQ files were aligned to the reference genome using Bismark^61^ (https://felixkrueger.github.io/Bismark/bismark/alignment/) with Bowtie2^62^ in directional mode and default parameters (--score_min L,0,−0.2, paired-end minimum/maximum insert size: -I 0, -X 500). CpG methylation levels were extracted from Bismark coverage files and imported into the methylKit^63^ (https://github.com/al2na/methylKit) R package for downstream analysis. To identify differentially methylated tiles, the tileMethylCounts function was applied with a window size of 1000 bp and a step size of 100 bp. Differentially methylated regions (DMRs) were detected using the calculateDiffMeth function with overdispersion = “MN” and adjust = “BH” for multiple testing correction. DMRs were ranked based on absolute methylation percentage differences, and statistical significance was defined as a false discovery rate (FDR) < 0.01 with an absolute methylation difference 25%. Results were visualized using Manhattan plots displaying the −log10-transformed p-values of individual tiling windows. For locus-level visualization on UCSC genome browser, base-resolution methylation information was extracted from Bismark BedGraph files and converted into a compatible format. Methylation levels were displayed as bar plots, where hypermethylated regions (50%–100% methylation) were shown in red on a scale from 0.5 to 1, and hypomethylated regions (0%–50% methylation) were shown in blue on a scale from −1 to −0.5.

### Platelet aggregation assay

CDPs generated as described above were harvested following the addition of prostaglandin I_2_ (PGI_2_; Cayman Chemical Company) and apyrase (Sigma-Aldrich) and gentle successive pipetting. Samples were centrifuged at 300 g for 5 minutes to remove intact cells, and CDPs were subsequently pelleted by centrifugation at 1,000 g for 10 minutes (low brake). Whole blood was collected into BD Vacutainer Citrate Tubes and centrifuged at 210 g for 15 minutes (low brake). Platelet-rich plasma (PRP) was collected, and modified Tyrode’s buffer (MTB) supplemented with PGI_2_ and apyrase was added. Platelets were then pelleted by centrifugation at 1,000 g for 10 min (low brake). at 800 rpm in a thermomixer for 5 minutes. Reactions were fixed by the addition of five volumes of 0.5% paraformaldehyde and analyzed on a Cytek Aurora flow cytometer. Data were analyzed using FlowJo.

CDPs and platelets were stained with CD31-Pacific Blue and PE, respectively, for 15 minutes at room temperature and washed with MTB. Cells were resuspended in MTB supplemented with CaCl_2_ (2 mM), human fibrinogen (0.1 mg/mL), CPD plasma (25% vol/vol), and 5 × 10^6^ platelets were combined with an equal number of CDPs from different editing. Agonists were added as indicated (ADP 10μM, TRAP 10 μM, U46619 0.25 μM, Ristocetin 1.5 mg/mL, Collagen 10 μg/mL), and samples were incubated at 37 °C with shaking at 800 rpm in a thermomixer for 5 minutes. Reactions were fixed by the addition of five volumes of 0.5% paraformaldehyde and analyzed on a Cytek Aurora flow cytometer. Data were analyzed using FlowJo.

### Quantification and statistical analysis

For experiments using human primary CD34^+^ HSPCs, data were obtained from at least two independent donors unless otherwise indicated. For xenotransplantation studies, seven recipient mice per group, including both male and female animals, were analyzed. For comparisons between two groups, statistical significance was assessed using a two-tailed unpaired t-test with Welch’s correction. For experiments involving more than two groups or multiple experimental conditions, one-way or two-way ANOVA was performed, followed by post hoc multiple-comparison correction. All data are presented as mean ± SD, significance is indicated as *P < 0.05, **P < 0.01, ***P < 0.001, or n.s. not significant.

## Supplementary Material

Supplement 1

Supplement 2

Supplement 3

Supplement 4

## Data Availability

All data and code will be released at the time of publication.
