# Supplementary material for: Epigenome editing of human hematopoietic stem cells enables sustained and reversible thrombosis prevention
Source: bioRxiv. 2026 Mar 29:2026.03.27.714536. Preprint. [Version 1] doi: 10.64898/2026.03.27.714536 (PMC13042045; doi:10.64898/2026.03.27.714536)
Supplement: Supplement 4 [file NIHPP2026.03.27.714536v1-supplement-4.pdf]

bioRxiv preprint doi: https://doi.org/10.64898/2026.03.27.714536; this version posted March 29, 2026. The copyright holder for this preprint (which was not certified by peer review) is the author/funder, who has granted bioRxiv a license to display the preprint in perpetuity. It is made available under aCC-BY 4.0 International license.

S1

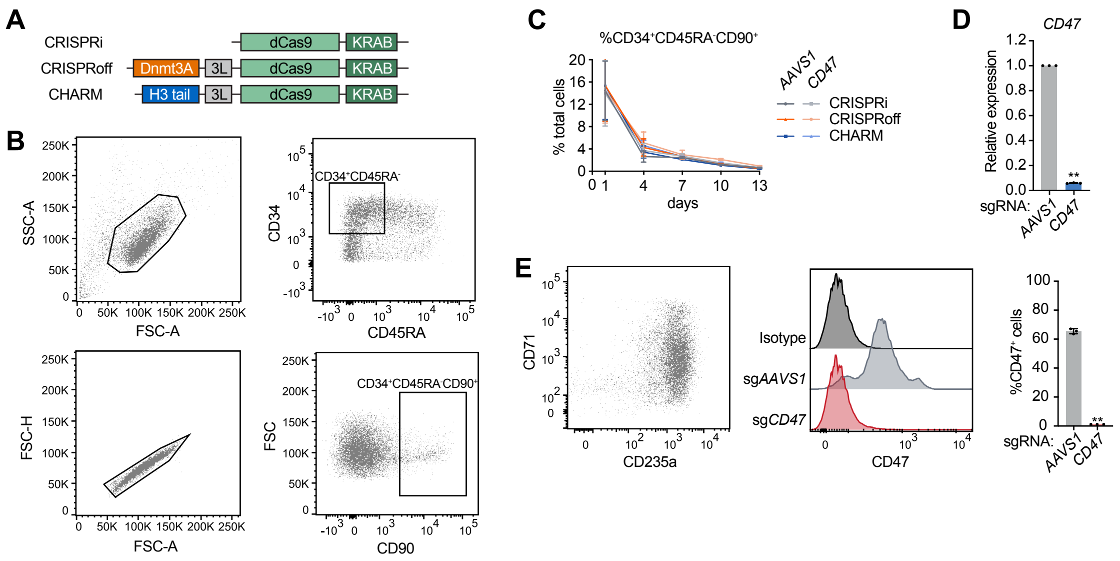

Supplemental Figure 1: (A) Schematic representation of CRISPRi, CRISPRoff, and CHARM constructs used in experiments. **(B)** Representative flow cytometry gating strategy used to define CD34⁺CD45RA⁻CD90⁺ cells within the HSPC culture. **(C)** Quantification of the percentage of CD34⁺CD45RA⁻CD90⁺ cells over time in HSPC cultures following editing with CRISPRi, CRISPRoff, or CHARM targeting the *CD47* promoter or *AAVS1* as a control. **(D)** Quantification of *CD47* mRNA expression measured by RT-qPCR in HSPCs 13 days after editing with CHARM targeting the *CD47* promoter or *AAVS1* as a control. **(E)** Representative flow cytometry plots showing erythroid differentiation profiles based on CD71 and CD235a expression following differentiation of CHARM-edited HSPCs. **(F)** Left, representative flow cytometry histograms of CD47 surface expression in erythroid cells differentiated from HSPCs edited with CHARM targeting the *CD47* promoter or *AAVS1* as a control. Right, quantification of the percentage of CD47⁺ cells. All data are presented as mean ± SD, significance is indicated as $*P < 0.05$, $**P < 0.01$, $***P < 0.001$, or n.s. not significant.

bioRxiv preprint doi: https://doi.org/10.64898/2026.03.27.714536; this version posted March 29, 2026. The copyright holder for this preprint (which was not certified by peer review) is the author/funder, who has granted bioRxiv a license to display the preprint in perpetuity. It is made available under aCC-BY 4.0 International license.

S2

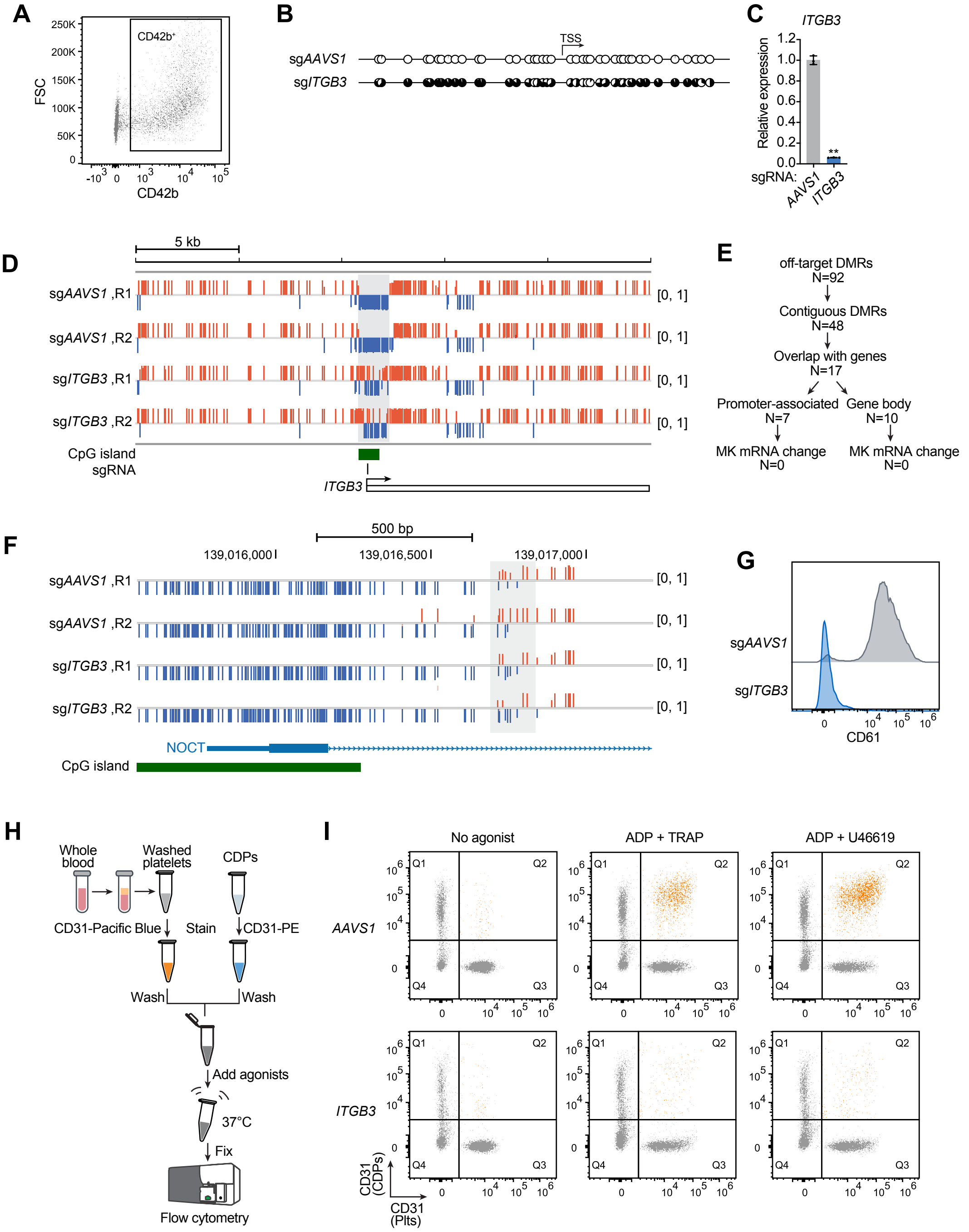

Supplemental Figure 2: (A) Representative flow cytometry gating strategy used to define CD42b⁺ mature megakaryocytes following differentiation of CHARM-edited HSPCs. **(B)** Targeted bisulfite sequencing displaying CpG methylation patterns near the *ITGB3* TSS in megakaryocytes differentiated from HSPCs edited with CHARM targeting the *ITGB3* promoter or *AAVS1* as a control. Each circle represents an individual CpG site; filled circles indicate methylated CpGs. **(C)** Quantification of *ITGB3* mRNA expression measured by RT-qPCR in differentiated megakaryocytes following CHARM targeting of the *ITGB3* promoter or *AAVS1* as a control. **(D)** Genome browser view comparing CpG methylation across a genomic window spanning the *ITGB3* locus in HSPCs edited with CHARM targeting the *ITGB3* promoter or *AAVS1* as a control. Red and blue marks denote CpGs with high (β-value > 0.5) or low (β-value < 0.5) DNA methylation, respectively. The annotated CpG island is shown in green, and the sgRNA target site is indicated. **(E)** Hierarchical annotation of off-target differentially methylated regions (DMRs) identified by WGBS in CHARM-edited HSPCs. The number of DMRs at each step is indicated. **(F)** Genome browser view comparing CpG methylation across a representative off-target locus (*NOCT*) in HSPCs edited with CHARM targeting the *ITGB3* promoter or *AAVS1* as a control. Tracks show two replicates per condition. Red and blue marks denote CpGs with high (β-value > 0.5) or low (β-value < 0.5) DNA methylation, respectively. The annotated CpG island is shown in green. The grey shaded region highlights the area differentially methylated. **(G)** Representative flow cytometry histograms of CD61 (*ITGB3*) surface expression in CDPs derived from HSPCs edited with CHARM targeting the *ITGB3* promoter or *AAVS1* as a control. **(H)** Schematic overview of the flow cytometry-based platelet aggregation assay used to assess the ability of CDPs to aggregate with donor platelets. **(I)** Representative flow cytometry plots assessing platelet-CDP aggregation under no agonist conditions or following stimulation with ADP + TRAP or ADP + U46619 for CDPs generated from HSPCs edited with CHARM targeting the *ITGB3* promoter or *AAVS1* as a control. All data are presented as mean ± SD, significance is indicated as **P* < 0.05, ***P* < 0.01, ****P* < 0.001, or n.s. not significant.

bioRxiv preprint doi: https://doi.org/10.64898/2026.03.27.714536; this version posted March 29, 2026. The copyright holder for this preprint (which was not certified by peer review) is the author/funder, who has granted bioRxiv a license to display the preprint in perpetuity. It is made available under aCC-BY 4.0 International license.

S3

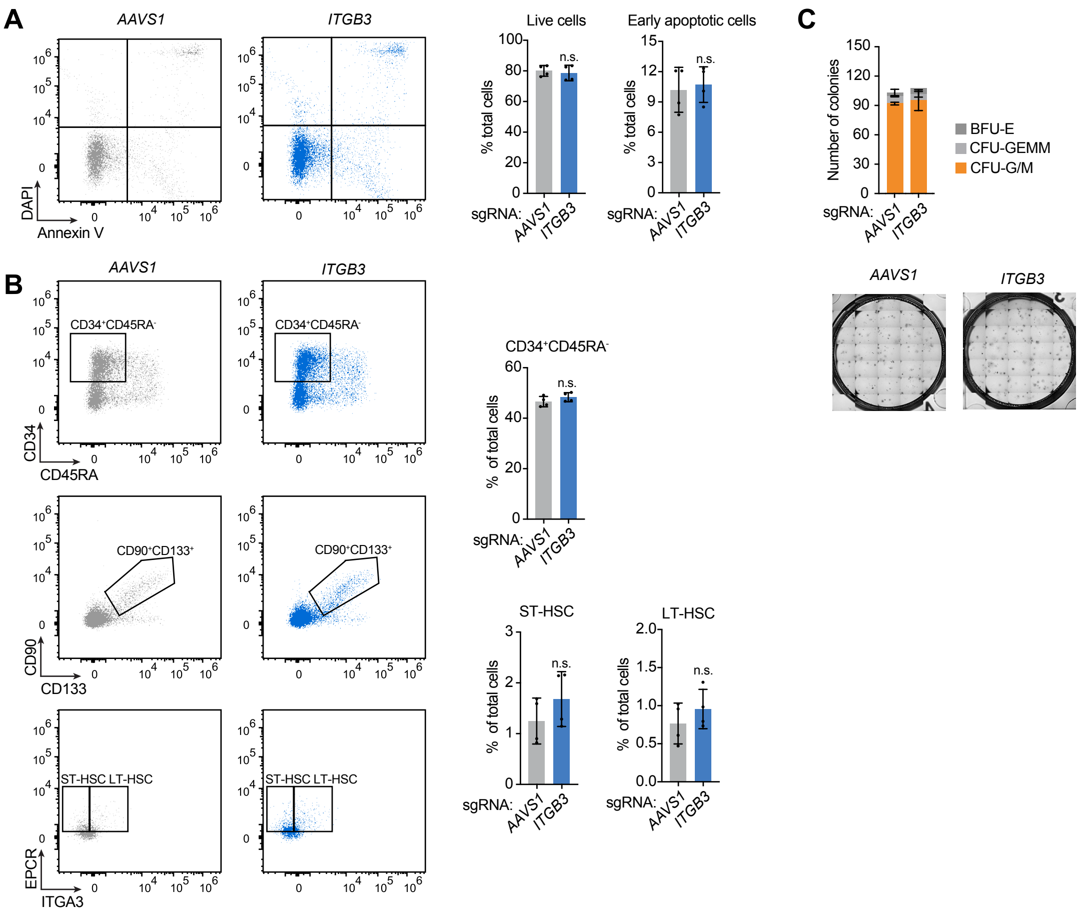

Supplemental Figure 3: (A) Left, representative flow cytometry plots of Annexin V and DAPI staining in HSPCs edited with CHARM targeting *ITGB3* or *AAVS1* as a control. Right, quantification of live cells and early apoptotic cells. **(B)** Left, representative flow cytometry plots of $CD34^+CD45RA^-$, LT-HSC ($CD34^+CD45RA^-CD90^+CD133^+EPCR^+ITGA3^+$) and ST-HSC ($CD34^+CD45RA^-CD90^+CD133^+EPCR^+ITGA3^-$) in HSPCs edited with CHARM targeting *ITGB3* or *AAVS1* as a control. Right, quantification of the indicated populations as a percentage of total cells. **(C)** Top, quantification of colony-forming units (BFU-E, CFU-GEMM, and CFU-G/M) generated from HSPCs edited with CHARM targeting the *ITGB3* or *AAVS1* as a control.

bioRxiv preprint doi: https://doi.org/10.64898/2026.03.27.714536; this version posted March 29, 2026. The copyright holder for this preprint (which was not certified by peer review) is the author/funder, who has granted bioRxiv a license to display the preprint in perpetuity. It is made available under aCC-BY 4.0 International license.

S4

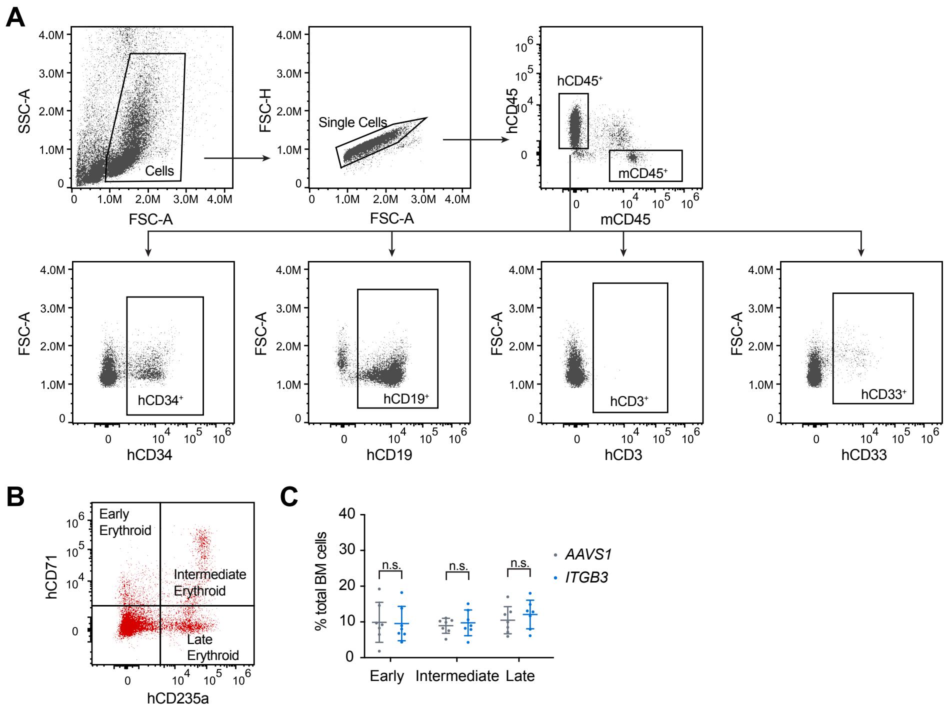

Supplemental Figure 4: (A) Representative flow cytometry gating strategy used to identify human hematopoietic populations in the bone marrow of transplanted NBSGW mice, including hCD45$^+$ cells and lineage-defined subsets (hCD34$^+$, hCD19$^+$, hCD3$^+$, and hCD33$^+$). **(B)** Representative flow cytometry profiles showing erythroid differentiation stages in the bone marrow of recipient mice, defined by hCD71 and hCD235a expression. **(C)** Quantification of early, intermediate, and late erythroid populations as a percentage of total bone marrow cells in mice transplanted with CHARM-edited HSPCs targeting the *ITGB3* ptomoter or *AAVS1* as a control. All data are presented as mean ± SD, significance is indicated as *$P < 0.05$, **$P < 0.01$, ***$P < 0.001$, or n.s. not significant.

bioRxiv preprint doi: https://doi.org/10.64898/2026.03.27.714536; this version posted March 29, 2026. The copyright holder for this preprint (which was not certified by peer review) is the author/funder, who has granted bioRxiv a license to display the preprint in perpetuity. It is made available under aCC-BY 4.0 International license.

S5

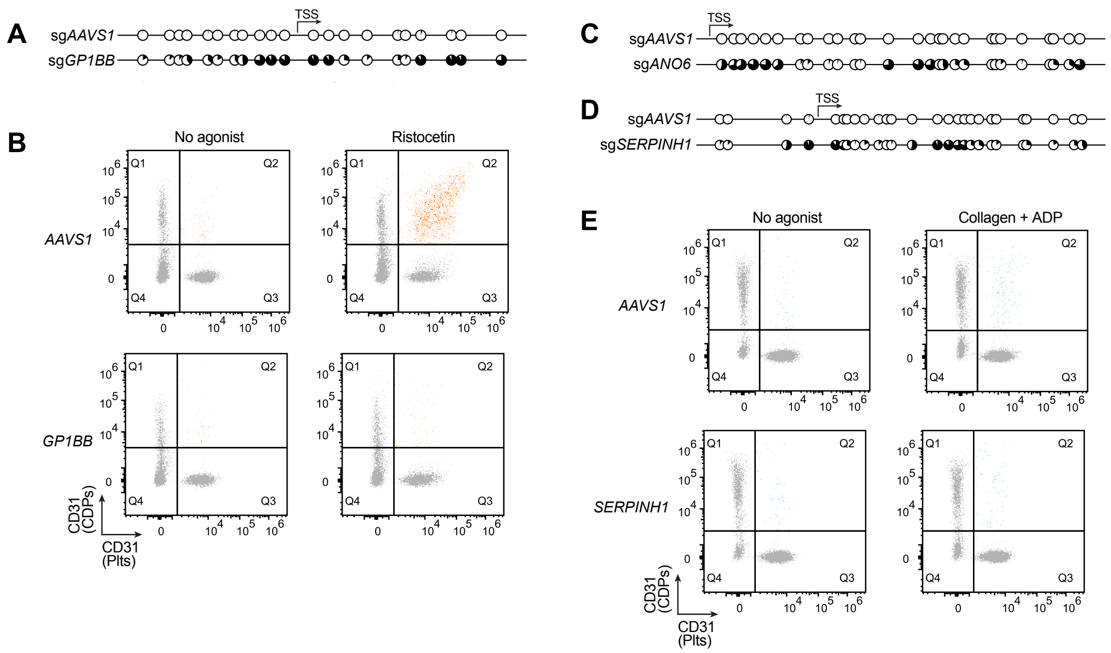

Supplemental Figure 5: (A) Targeted bisulfite sequencing displaying CpG methylation patterns near the *GP1BB* TSS in megakaryocytes differentiated from HSPCs edited with CHARM targeting the *GP1BB* promoter or *AAVS1* as a control. Each circle represents an individual CpG site; filled circles indicate methylated CpGs. **(B)** Representative flow cytometry plots assessing platelet-CDP aggregation under no agonist conditions or following stimulation with ristocetin for CDPs generated from HSPCs edited with CHARM targeting the *GP1BB* promoter or *AAVS1* as a control. **(C–D)** Targeted bisulfite sequencing displaying CpG methylation patterns near the *ANO6* (C) and *SERPINH1* (D) TSSs in megakaryocytes differentiated from HSPCs edited with CHARM targeting the respective promoters or *AAVS1* as a control. Each circle represents an individual CpG site; filled circles indicate methylated CpGs. **(E)** Representative flow cytometry plots assessing platelet-CDP aggregation under no agonist conditions or following stimulation with collagen + ADP for CDPs generated from HSPCs edited with CHARM targeting the *SERPNH1* promoter or *AAVS1* as a control.
